# Neoadjuvant FOLFOX 4 versus FOLFOX 4 with Cetuximab versus immediate surgery for high-risk stage II and III colon cancers: a multicentre randomised controlled phase II trial – the PRODIGE 22 - ECKINOXE trial

**DOI:** 10.1186/s12885-015-1507-3

**Published:** 2015-07-10

**Authors:** Mehdi Karoui, Anne Rullier, Alain Luciani, Franck Bonnetain, Marie-Luce Auriault, Antony Sarran, Geneviève Monges, Hervé Trillaud, Karine Le Malicot, Karen Leroy, Iradj Sobhani, Armelle Bardier, Marie Moreau, Isabelle Brindel, Jean François Seitz, Julien Taieb

**Affiliations:** 1 Assistance Publique-Hôpitaux de Paris, Pitié-Salpêtrière University Hospital, Department of Digestive and Hepato-Pancreato-Biliary Surgery, University Institute of Cancerology (Paris VI), Pierre & Marie Curie University (Paris VI), 47-83 Boulevard de l’Hôpital, 75013 Paris, France; 2 FFCD (Fédération Francophone de Cancérologie Digestive), Dijon, France; 3 Department of Pathology, Pellegrin University Hospital, Bordeaux, France; 4 Assistance Publique-Hôpitaux de Paris, Department of Radiology, Henri Mondor University Hospital, Paris XII university, Créteil, France; 5 Department of Medical Oncology and public health, Centre Hospitalier Régional Universitaire Hôpital Jean Minjoz, Besançon, France; 6 Assistance Publique-Hôpitaux de Paris, Department of Pathology, Henri Mondor University Hospital, Créteil, France; 7 Department of Radiology, Institut Paoli Calmettes, Marseille, France; 8 Department of Pathology, Institut Paoli Calmettes, Marseille, France; 9 Department of Radiology, St André University Hospital, Bordeaux, France; 10 Assistance Publique-Hôpitaux de Paris, Department of Gastroenterology, Henri Mondor University Hospital, Créteil, France; 11 Assistance Publique-Hôpitaux de Paris, Department of Pathology, Pitié-Salpêtrière University Hospital, Paris, France; 12 Assistance Publique-Hôpitaux de Paris, Département de la Recherche Clinique et du Développement (DRCD), Paris, France; 13 Assistance Publique-Hôpitaux de Paris, Department of Digestive Oncology, European Georges Pompidou – Paris Descartes University, Paris, France

**Keywords:** Colon cancer, Locally advanced disease, Neoadjuvant chemotherapy, FOLFOX, Cetuximab, Randomized phase II trial

## Abstract

**Background:**

In patients with high risk stage II and stage III colon cancer (CC), curative surgery followed by adjuvant FOLFOX-4 chemotherapy has become the standard of care. However, for 20 to 30 % of these patients, the current curative treatment strategy of surgical excision followed by adjuvant chemotherapy fails either to clear locoregional spread or to eradicate distant micrometastases, leading to disease recurrence. Preoperative chemotherapy is an attractive concept for these CCs and has the potential to impact upon both of these causes of failure. Optimum systemic therapy at the earliest possible opportunity may be more effective at eradicating distant metastases than the same treatment given after the delay and immunological stress of surgery. Added to this, shrinking the primary tumor before surgery may reduce the risk of incomplete surgical excision, and the risk of tumor cell shedding during surgery.

**Methods/Design:**

PRODIGE 22 - ECKINOXE is a multicenter randomized phase II trial designed to evaluate efficacy and feasibility of two chemotherapy regimens (FOLFOX-4 alone and FOLFOX-4 + Cetuximab) in a peri-operative strategy in patients with bulky CCs. Patients with CC deemed as high risk T3, T4 and/or N2 on initial abdominopelvic CT scan are randomized to either colectomy and adjuvant chemotherapy (control arm), or 4 cycles of neoadjuvant chemotherapy with FOLFOX-4 (for *RAS* mutated patients). In *RAS* wild-type patients a third arm testing FOLFOX+ cetuximab has been added prior to colectomy. Patients in the neoadjuvant chemotherapy arms will receive postoperative treatment for 4 months (8 cycles) to complete their therapeutic schedule. The primary endpoint of the study is the histological Tumor Regression Grade (TRG) as defined by Ryan. The secondary endpoints are: treatment strategy safety (toxicity, primary tumor related complications under chemotherapy, peri-operative morbidity), disease-free and recurrence free survivals at 3 years, quality of life, carcinologic quality and completeness of the surgery, initial radiological staging and radiological response to neoadjuvant chemotherapy, and the correlation between histopathological and radiological response. Taking into account a 50 % prevalence of CC without *RAS* mutation, accrual of 165 patients is needed for this Phase II trial.

**Trial Registration:**

NCT01675999 (ClinicalTrials.gov)

## Background

With about 1 million new cases in developed countries annually and 500 000 annual deaths, colon cancer (CC) is the second leading cause of cancer death in Western countries and a significant public health issue [1]. Curative treatment of CC, possible in nearly 80 % of cases, is based on carcinologic surgical resection. For stage III CC (any T/N1-2/M0), adjuvant FOLFOX-4 chemotherapy for 6 months is the standard of care. In the MOSAIC trial [2] that compared adjuvant LV5FU2 with FOLFOX-4 in 2246 stage II and III CC patients, there was a significant increase in disease-free survival at 3 years in the FOLFOX-4 group (78.2 % vs. 72.9 %, for all patients combined; 72.2 % vs. 65.3 % for the stage III subgroup). The 5 % difference in events at 3 years (26.1 % vs. 21.1 %) resulted in a hazard ratio (HR) of 0.77 corresponding to a 23 % risk reduction for patients receiving oxaliplatin. These results were confirmed at 4- and 6-years [3, 4]. For stage II CC (T3-4/N0/M0), the benefit of adjuvant chemotherapy remains controversial and there is a significant heterogeneity in patients outcome within this stage. In the MOSAIC study, high risk stage II patients defined as N0/M0 patients with a T4 primary tumor, bowel obstruction, tumor perforation, poorly differentiated tumor, tumors with satellite venous or lymphatic invasion, or less than 10 lymph nodes examined, a non-significant gain of 3 % in disease-free survival at 3-years was observed for the FOLFOX-4 arm compared with the LV5FU2 arm (HR: 0.72 [0.48-1.08]) [5]. The published QUASAR 2 study [6] compared adjuvant 5-FU plus Folinic Acid ± Levamisole with no adjuvant chemotherapy in 3,239 patients with colorectal cancer (92 % stage II in a mixed population of 71 % colon and 29 % rectal cancer). The results showed an absolute gain of 2.9 % in overall survival at 5-years for the chemotherapy group for the entire population (p = 0.02, and for stage II, p = 0.04). The absolute benefit of adjuvant chemotherapy in stage II CC patients is generally considered between 2 and 5 %. Therefore, it is currently recommended that adjuvant therapy shall be discussed on a case-by-case basis, considering the risk-benefit ratio for each patient resected from a stage II disease, with strong consideration given to patients with high risk stage II CC as defined previously [7, 8].

The 20 to 30 % risk of local or distant recurrence observed in patients with stage II/III, non-metastatic CC operated in a curative intent and receiving adjuvant chemotherapy reflects the relative failure of such a strategy to prevent the risk of locoregional spread of tumor cells or eradicate distant micrometastases. Several reasons may explain this failure: the delayed start (up to 4 months) of chemotherapy after the initial diagnosis and the rapid doubling time of colorectal metastases, which may progress during this time interval without chemotherapy [9]. The stimulation of growth factors related to the operation and the immunosuppression induced during the immediate postoperative period are two other factors that may promote the growth of micrometastases and tumor progression in the postoperative setting [10–12]. The initiation of a neoadjuvant chemotherapy in these patients could improve prognosis by controlling these potential deleterious factors and improving the completeness and the quality of the cancer surgery by local "down-staging,” and by eradicating circulating micrometastases. Neoadjuvant chemotherapy could also test the response of the tumor and thus its chemo-sensitivity; and when appropriate, adjuvant chemotherapy may be adapted.

The potential benefit of such a peri-operative strategy in stage II/III CC is strengthened by: i) recent advances in radiology, which allows a good prediction of tumor stage (wall penetration and nodal involvement) prior to surgery [13, 14]; ii) the histological response observed in primary colon tumors treated by systemic chemotherapy [15] and iii) the demonstrated benefit of combining pre- and postoperative chemotherapy and/or radiotherapy in various gastrointestinal tumors (oesophageal, gastric, rectal and colorectal liver metastases) by several randomized trials [16–18].

## Methods/Design

### Protocol overview

The Prodige 22 - ECKINOXE trial is a multicenter randomized phase II trial designed to evaluate efficacy (response rate) and feasibility (safety, tolerability) of two chemotherapy regimens (FOLFOX-4 alone and FOLFOX-4 + Cetuximab) in a peri-operative setting in patients with stage II/III CC. Control arm includes patients for whom standard treatment comprises surgery followed by adjuvant FOLFOX-4 chemotherapy. This phase II study will assess the feasibility of a peri-operative strategy in these patients and the impact of adding cetuximab in this setting for patients with *RAS* wild type tumors.

A double central, independent and blinded review of imaging and pathology is planned. All eligible patients will be randomized to either surgery alone or neoadjuvant chemotherapy with simplified FOLFOX-4. Patients randomized to the simplified FOLFOX-4 arm will be randomized to receive Cetuximab or not if they are *RAS* WT

**Fig. 1:**
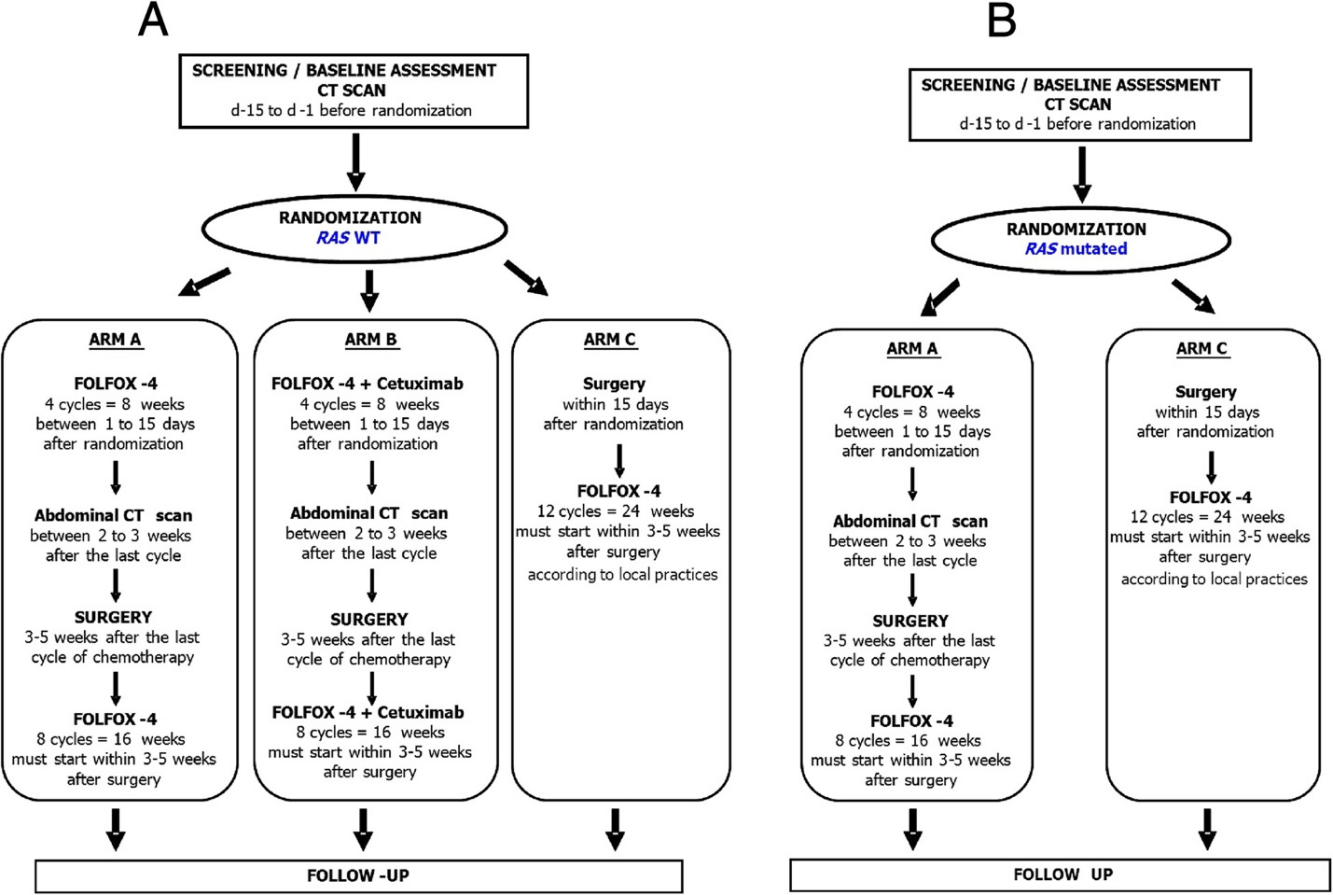
Protocol overview. Temporal sequence of trial conduct in patients with *RAS* WT colon tumor (1A) or RAS mutated colon tumor (1B)

Thus based on the *RAS* status of the primary tumor the design will be: Arm A vs. Arm B vs. Arm C for *RAS* WT patients and Arm A vs. Arm C for those *RAS* mutated (Fig. 1)

Arm A: simplified FOLFOX-4 alone every 2 weeks for 4 cycles, followed by colectomy, followed by simplified FOLFOX-4 alone every 2 weeks for 8 cycles.

Arm B: Cetuximab plus simplified FOLFOX-4 every 2 weeks for 4 cycles, followed by colectomy, followed by Cetuximab plus simplified FOLFOX-4 every 2 weeks for 8 cycles.

Arm C (control arm): Colectomy followed by simplified FOLFOX-4 alone every 2 weeks for 12 cycles. Adjuvant chemotherapy will be given to all patients with stage III CC and according to the local practices for stage II cancer.

### Inclusion criteria

For inclusion in the study, all of the following inclusion criteria must be fulfilled: (i) pathologically confirmed colon adenocarcinoma (≥15 cm from the anal verge); (ii) assessment of *RAS* status of the primary colon cancer on biopsies (WT or mutated); (iii) CC classified by abdominal CT scan: high risk T3 (disruption of muscle wall and extension into pericolic fat with more than 5 mm protrusion into adjacent mesenteric fat) - T4 (penetration within adjacent organs) and/ or N2 (more than 3 clustered lymph nodes above 1 cm in shortest diameter); (iv) non metastatic CC (lung, liver, peritoneal); (v) non complicated primary tumor (obstruction, perforation, bleeding). Patients with obstructive CC and treated by defunctioning stoma can be included; (vi) absence of synchronous colorectal cancer ; (vii) age ≥ 18 years et ≤ 75 years; (viii) ECOG performance status 0–1 ; (ix) no prior chemotherapy or abdominal or pelvic irradiation; (x) life expectancy of ≥ 5 years; (xi) no history of colorectal cancer; (xii) patients with childbearing potential should use effective contraception during the study and the following 6 months; (xiii) laboratory data including : white blood cell count ≥ 3 × 10^9^/L with neutrophils ≥ 1.5 × 10^9^/L, platelet count ≥ 100 × 10^9^/L, hemoglobin ≥ 9 g/dL (5,6 mmol/l), total bilirubin ≤ 1.5 × ULN (upper limit of normal), ASAT and ALAT ≤2.5 × ULN, Alkaline phosphatase ≤1.5 × ULN, serum creatinine ≤ 1.5 × ULN; (xiv) signed written informed consent obtained prior to any study specific screening procedures.

### Exclusion criteria

Patients are not eligible for this study if any of the following exclusion criteria apply: (i) contra-indication to iodinated contrast medium injection including allergy to iodinated contrast medium and renal insufficiency proscribing iodinated injection; (ii) rectal cancer located within 15 cm from the anal verge by endoscopy or under the peritoneal reflection at surgery or having received radiation therapy prior to surgery; (iii) complicated primary CC (obstruction, bleeding, perforation); (iv) synchronous colorectal cancer; (v) metastatic spread at baseline assessment (lung, liver, peritoneal); (vi) history or current evidence on physical examination of central nervous system disease or; (vii) peripheral neuropathy ≥ grade 1 Common Toxicity Criteria for Adverse Events (CTCAE) v.3.0; (viii) known hypersensitivity reaction to any of the components of study treatments; (ix) presence of inflammatory bowel disease; (x) HNPCC syndrome or polyposis; (xi) major surgical procedure, open biopsy or significant traumatic injury within 28 days prior to study treatment start. Incompletely healed wounds or anticipation of the need for major surgical procedure during the course of the study; (xii) clinically relevant coronary artery disease or history of myocardial infarction in the last 12 months, or high risk of uncontrolled arrhythmia; (xiiii) pregnancy (absence to be confirmed by ß-hCG test) or breast-feeding period; (xiv) previous malignancy in the last 5 years; (xv) medical, geographical, sociological, psychological or legal conditions that would not permit the patient to complete the study or sign informed consent; (xvi) any significant disease which, in the investigator’s opinion, would exclude the patient from the study.

### Endpoints

By evaluating two chemotherapy regimens in patients with *RAS* WT tumor (FOLFOX-4 vs. FOLFOX-4 – Cetuximab), the present trial will determine which neoadjuvant regimen is the most efficient in terms of response rate in patients with non metastatic locally advanced CC.

**Fig. 2:**
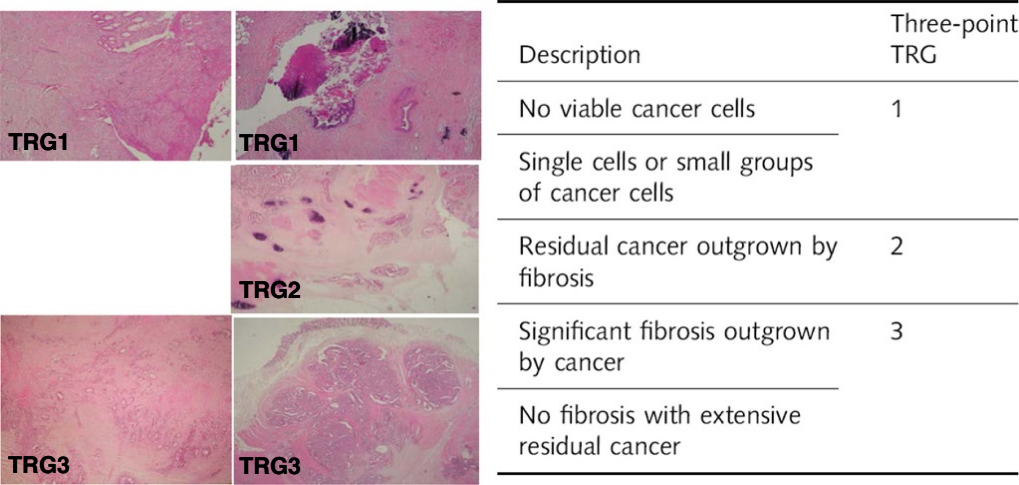
The Tumor Regression Grade (TRG), as simplified to three categories by Ryan (19). TRG1: Major response; TRG2: Intermediate response; TRG3: No response

The primary objective is to assess the histopathological response to neoadjuvant chemotherapy measured by the Tumor Regression Grade (TRG), as simplified to three categories by Ryan [19]. The TRG-Ryan is based on the relationship between fibrosis and the amount of residual tumor cells is defined as follows (Fig. 2). The histopathological finding of fibrous stroma containing few viable tumor cells can be naturally present in almost 10 % of CCs not treated with chemotherapy prior to surgery [15]. Therefore, this parameter represents an objective measure of evaluation in the control (no pre-operative chemotherapy) and study (neoadjuvant chemotherapy) arms. This limits the risk of wrongly concluding therapy efficacy, thereby meeting the methodological standards of a Phase II randomized study. The histopathological response to neoadjuvant chemotherapy will be centrally assessed by 2 of the 3 members of our Central Review Committee of Pathologists who will be blinded to the patients’ treatment. The objective of the blinded comparison of TRG by two independent pathologists is to assess: (i) the inter-observer reproducibility of this grading system in CC after neoadjuvant chemotherapy; (ii) the specificity of histological "regression" criteria by comparing, without knowing the treatment arms, the histological features of tumors operated on immediately versus that of tumors treated by neoadjuvant chemotherapy.

Secondary endpoints are the following: (i) postoperative morbidity (occurring within 60 postoperative days) graded 0 to 5 according to the Clavien-Dindo classification [20]. If grade 3 or 4 complications occur at a rate ≥ 10 % in either neoadjuvant chemotherapy arm, the arm will be declared hazardous (see section statistical analysis); (ii) the safety of neoadjuvant therapy will be assessed by the systematic collection of toxicity data related to chemotherapy, the total number of cycles administered pre-operatively (documenting reasons for early termination), and the systematic collection of GI symptoms. In addition, complications related to the in situ tumor that could delay the date of surgery or require emergency colonic surgery or stenting, will be documented. These parameters will be evaluated after administration of the first cycle of neoadjuvant chemotherapy; (iii) Regression-free survival at 3-years defined as the time interval between the date of randomization and the date of first recurrence (local, regional or metastatic) or date of death (all causes) if no recurrence; (iv) Disease-free survival at 3 years defined as the time interval between the date of randomization and the date of first recurrence (local, regional or metastatic), or second cancer, or the date of death without recurrence (all causes) and date of second cancer; (iv) Quality of life (evaluated using the EORTC quality of life (QoL) QLQ-C30 and QLQ-CR29); Quality of life will be assessed at the inclusion, after neoadjuvant chemotherapy, at 1 month after surgery and every 6 months thereafter. The QLQ-C30 consists of 30 items with both multi-items scales and single item measures. The internal validation of QLQ-C30 allowed to identify 15 scales and to generate 15 scores: 5 scores of functional scale (physical functioning, role functioning, cognitive functioning, emotional functioning, social functioning), a global health status scare, a financial difficulties scale and 8 symptoms scale (fatigue, nausea/ vomiting, pain, dyspnoea, insomnia, appetite loss, constipation, diarrhoea). These scores vary from 0 (worse) to 100 (better) for functional and global health scale and from 0 (better) to 100 (worse) for symptoms scales. Targeted QoL dimensions for this trial are Global Health, physical and emotional functioning, fatigue and pain of QLQ-30 as well as dimensions of CR29. A 5 points difference in QoL is considered as minimal clinically important difference (MCID). Time to QoL deterioration will be defined as time interval between randomization and first decrease in QoL score greater or equal to 5 points. Patients without QoL decrease greater or equal to 5 points will be censored at the last follow-up. (v) Quality and completeness of the surgical excision evaluated on pathological criteria (number of lymph nodes examined, the completeness of removed mesocolon, surgical margins); (vi) radiological staging and radiological response. The initial radiological staging based on abdominopelvic CT scan will be compared with the histopathological pTNM staging in the control group. CT scan images will be interpreted by the individual study centers, and reviewed by 2 of the 3 members of our Central Review Committee of Radiology. This dual independent reading will assess inter-observer reproducibility of the radiological response to treatment with a test of concordance. The radiological response will be defined by RECIST (v1.1) [21] on CT scan and evaluated in the neoadjuvant chemotherapy arms. These criteria will be applied to the primary colon tumors (largest diameter) and lymph nodes (smallest diameter). The sum of the diameters of the target lesions will be determined on the pretreatment CT scan (initial staging) and compared with the sum of these measures on the reevaluation CT scan (performed within 3 weeks after the completion of neoadjuvant chemotherapy). Interpretation of the reevaluation CT scan will be centralized and carried out independently by 2 of the 3 members of our Central Review Committee of Radiologists blinded to the pretreatment CT scan staging. This dual independent reading will assess inter-observer reproducibility of RECIST criteria applied to CC, and determine if there is a difference in radiological response between the two chemotherapy regimens under investigation in the study; (vii) Correlation between histopathological and radiological response. The radiological response of the primary tumor as defined by RECIST v1.1 criteria (taken for target lesion, only the primary tumor) will be compared with the histopathological response as defined by TRG-Ryan. Evaluation of this correlation will be centralized and carried out by the Central Review Committee of Radiology and Pathology; (viii) The 5-point regression grading system [22] adapted to CC by our team in a pilot study [15], will also be evaluated in this trial. This assessment will be centralized and carried out independently and blindly (without knowing the treatment arms) by 2 of the 3 members of our Central Review Committee of Pathology. This assessment will determine the inter-observer reproducibility of this grading system in CC after chemotherapy and to assess the specificity of histological "regression" criteria using this grading system. Comparison of the reproducibility (test of concordance) of the two systems of grading regression (TRG-Ryan and TRG-Mandard) will identify the most appropriate system for assessing response to chemotherapy in CC.

### Randomisation

After completion of all the screening evaluations (all the inclusion criteria satisfied and none of the exclusion criteria apply) and signature of the informed consent(s), all eligible patients will be randomly assigned by the randomisation center of the French Federation of Digestive Oncology (FFCD) to one of the treatment arms according to the RAS status: *RAS* WT patients will be randomised between 3 treatment arms (Arm A vs. Arm B vs. Arm C) and those mutated for RAS between 2 treatement arms (Arm A vs. Arm C). The randomization will be stratified according to the investigator center, T stage (T1/2/3 vs. T4) of the primary tumor on the pretreatment CT scan and N stage (N0-N1 vs. N2) of the primary tumor on the pretreatment CT scan. The screening/baseline assessment will take place within the 3 weeks prior to randomization, randomization will be done between 4 and 8 weeks after surgery and the maximum time between randomization and treatment start will be 15 days.

### Pre-therapeutic work-up

The screening visit must be performed within 3 weeks before the randomisation. During this visit, inclusion and exclusion criteria are checked and validated. The complete pre-therapeutic work-up includes a physical examination, standard laboratory tests with CEA level, complete colonoscopy with biopsies and a CT scan of the thorax, abdomen and pelvis. Clinical tumoural staging (cTNM) will be based on the data obtained from CT scan. The determination of the *RAS* status (*KRAS* and *NRAS* mutations located within the exons 2, 3 and 4) of the primary tumor will be performed from biopsies obtained during the diagnostic colonoscopy, by each centers at the “plateformes de génétique somatique des tumours” labelled by the National Institut of Cancer (INCa). Once baseline assessments are completed, check and validation of inclusion and exclusion criteria for the study will be performed at the oncological multidisciplinary meeting of each center.

### Treatment methods

The treatment protocols are standardized with regard to radiological and pathological parameters, based on the recommendations of I’INCa, the FFCD, the French Society of Pathology, the French Society of Digestive Endoscopy and the French Society of Digestive Surgery. Treatment application depends on the *RAS* status of the primary tumor (Fig. 1) and must start within 14 days after the randomisation.

Neoadjuvant chemotherapy requires an implantable subcutaneous venous access and will be administrated for 4 cycles. The following assessments will be performed prior to each cycle (every two weeks) before chemotherapy administration: documentation of concomitant medication and concurrent procedures; physical examination; vital signs and ECOG performance status; blood sampling for hematology and clinical biochemistry and recording of adverse events since last cycle (including primary tumor related symptoms or complications). The end of treatment visit must be performed 15 days after the 4^th^ cycle administration.

#### FOLFOX-4 chemotherapy

The simplified FOLFOX-4 (Oxaliplatine, ELOXATINE^R^ (85 mg/m^2^), with simplified LV5FU2) is administrated intravenously over 48 hours once every two weeks (i.e. on d1/d2, d15/d16, d29/d30 etc.). The FOLFOX-4 regimen will be administrated as follows: Oxaliplatin will be administered as an 85 mg/m^2^ intravenous infusion over 2 hours (on day 1 only) concomitantly with LV, as a 400 mg/m^2^ infusion over 2 hours, followed by 5- fluorouracil (5-FU), given as a 400 mg/m^2^ bolus injection (over 10 minutes), and then as a 2400 mg/m^2^ continuous infusion over 46 hours. Cycle length is 2 weeks comprising 48 hours of infusion and 12 days of rest. These cycles must be repeated every second week. A total of 12 cycles (4 cycles preoperatively, 8 cycles postoperatively) will be administrated to all patients in Arm A and B. In Arm C (control Arm), only patients with stage III CC (on pathological examination) will receive 12 cycles of adjuvant chemotherapy. For those with stage II CC, the decision of adjuvant chemotherapy in this Arm will be will be done according to the investigator discretion.

#### FOLFOX-4 + Cetuximab chemotherapy

Patients in Arm B only will receive Cetuximab according the same schedule of the FOLFOX chemotherapy, i.e. once every two weeks (on d1, d14, d28, etc.…). Cetuximab will always be administered first, i.e. the Cetuximab infusion should be completed one hour before chemotherapy begins. The Cetuximab dose must always be based on the body surface area (BSA). There is no restriction for Cetuximab in patient with a BSA > 2.0 m^2^. The dosage and administration procedure for Cetuximab is as follows:

- The first infusion is 500 mg/m^2^. As prophylaxis to reduce the risk of an allergic/hypersensitivity reaction, it is mandatory to pretreat the patient as specified below. Cetuximab is administered as follows: the volume is administered over 120 minutes (maximum rate of 5 mL/min). Saline solution (0.9 %) is used to flush the line at the end of the infusion. Vital signs are checked before, during, immediately after and 1 hour after the end of the infusion.

The subsequent weekly doses are 500 mg/m^2^. Cetuximab is administered as follows: the volume is administered over 60 minutes (maximum rate of 10 mL/min). Saline solution (0.9 %) is used to flush the line at the end of the infusion.

Surgery will be performed 3 to 5 weeks after the last cycle of neoadjuvant chemotherapy (in arms A and B) or within 14 days after the randomisation. The anesthetic evaluation, patient’s information regarding the operative procedure and the organization of the hospitalization will be performed according to the local practices of each investigator center. The colectomy will be performed by laparotomy or laparoscopy (investigator discretion) with respect of the oncological quality criteria of resection. Per and postoperative data will be collected and reported in specific forms.

Adjuvant chemotherapy will be administrated for all patients included in arms A (FOLFOX-4) and B (FOLFOX-4 + Cetuximab). Eight additional cycles will be administrated (for a total of 12 cycles) according the same regimen. In Arm C (control Arm), only patients with stage III CC (on pathological examination) will receive 12 cycles of adjuvant chemotherapy. For those with stage II CC, the decision of adjuvant chemotherapy in this arm will be will be done according to the investigator's discretion.

### Data collection and follow-up

For all patients, follow-up assessment will be performed until recurrence and/or death. For the study, the recurrence free survival will be assessed at 3 years. For patients included in the present trial, the follow-up will be systematic and performed thereafter this period according to the good clinical practices. The follow-up schedule and investigations proposed for the present study are those recommended by the FFCD [8].

Every 6 months, the following investigations will be performed: tumor assessment, CEA measurement, survival status, additional cancer therapy. Moreover, the following assessments will be performed during the follow-up: colonoscopy 3 years after surgery then every 3 to 6 years, assessment of neurological toxicity, and assessment of electrolytes (magnesium, calcium, potassium) at the second follow up visit (12 months after surgery) if still abnormally decreased at end of treatment assessment.

### Statistical analysis and sample size

Patients RAS Wild Type will be randomized between arm A, B and C whereas RAS mutated patients will be only randomized between arm A and arm C. According to Simon (Optimax) 2 steps design, it will be required to include 33 patients/ arm (with unilateral alpha type one error of 5 % and a power of 95 %) to test following hypotheses: (i) H_0_: an histological response (TRG1-Ryan) rate of 10 % is uninteresting; (ii) H_1_: an histological response (TRG1-Ryan) higher than 10 % is required to rate pursue investigations in phase III trial. A response rate of 35 % is expected.

At the first step (interim analysis), after inclusion of the first 13 patients in each arm, if the number of histological response (TRG1-Ryan) is ≤ 1, inclusion in this arm will be stopped and if the number of histological response (TRG1-Ryan) is ≥ 2, inclusion of the next 20 patients will be done. Calculated power at the interim analysis is 97 %.

At the 2^nd^ step (final analysis), after inclusion of 33 patients in each arm, if the number of histological response (TRG1-Ryan) is ≤ 6, this arm will be declared uninteresting for further investigations in phase III trial and if the number of histological response (TRG1-Ryan) is ≥ 7, this arm will be declared interesting for further investigations in phase III trial. At the second step calculated power will be 96.3 % and alpha type one error will be 2 %. In control arm no interim analysis is plan for efficacy. Then in this arm, at the second step, calculated power will be 97.2 % and alpha type one error will be 4.2 %. If ≥ 10 % of complications ≥ grade III (Clavien and Dindo classification) within 60 postoperative days are reported, this arm will be declared unsafe.

Interim and final results of this phase II study will be reviewed by an independent data monitoring committee (IDMC). Trial will be pursued in phase III with 2 or 3 arms according to IDMC recommendations. Control arm (no neoadjuvant treatment) will be kept as control arm in phase III whatever the results of phase II study. Required number of patients is 33 × 3 = 99 patients for RAS WT patients and 33 × 2 = 66 for those with RAS mutated. Taking into account prevalence of RAS WT patients of 50 %, it will be necessary to screen 192 patients to obtain 99 patients RAS WT and 66 patients mutated for RAS with a minimal probability of 90 %.

### Participating centres

Fifty seven French centres will participate in the study: Amiens University Hospital, Caluire et Cuire Hospital in Lyon, University Hospital of Clermont-Ferrand, General Hospital Center of Orléans, European Georges Pompidou University Hospital in Paris, Claude Huriez University Hospital in Lille, North University hospital of Bordeaux, University Hospital of Nîmes, Purpan University Hospital in Toulouse, Paoli Calmettes Cancer Institute in Marseille, Henri Mondor University Hospital in Créteil, North University Hospital in Marseille, Saint-Louis University Hospital in Paris, Pitié Salpêtrière University Hospital in Paris, Ambroise Paré University Hospital in Boulogne-Billancourt, St André University Hospital in Bordeaux, General Hospital Center in Beauvais, Croix-Rousse University Hospital in Lyon, Beaujon University hospital in clichy, Pessac University Hospital of Bordeaux, the University Hospital in Strasbourg, Pontchaillou University Hospital in Rennes, General hospital in Bézier, Robert Debré University Hospital in Reims, Avicenne University Hospital in Bobigny, Cochin University Hospital in Paris, St Anne Clinic in Strasbourg, University Hospital of Nantes, Jean Mermoz Private Hospital in Lyon, University Hospital in Grenoble, La Timone University Hospital in Marseille, Saint Antoine University Hospital in Paris, General Hospital of Pau, Paul Strauss Cancer Institute in Strasbourg, University Hospital of Dijon, Saint Joseph Private Hospital in Paris, University Hospital of Rouen, Rangueil University Hospital in Toulouse, Sainte Barbe Clinic in Strasbourg, Sainte Catherine Institut in Avignon, The Asclepios House Clinic in Avignon, General Hospital of Bayonne, Eugene Marquis Cancer Institut of Rennes, Intercommunal Hospital Center of Creteil, General Hospital of Mont de Marsan, Bichat University Hospital in Paris, Grand Sud Polyclinic of Nîmes, Kenval Polyclinic of Nîmes, Valdegour Clinic Oncogard Center of Nîmes, General Hospital of Boulogne Sur Mer, University Hospital of Poitiers, University Hospital of Brest, Saint Joseph-Saint Luc Hospital in Lyon, Synergia Polyclinic in Carpentras, General Hospital of Avignon, University Hospital of Saint Etienne.

### Ethics and safety

This study protocol was approved by the Institutional Review Board, the Ile de France IX ethic committee on November 2011 and the AFSSAPS (Agence Française de Sécurité Sanitaire des Produits de Santé) on September 2011 under the registration number A110936-41. The institutional promoter is the Assistance Publique Hôpitaux de Paris, France. The trial has been registered on ClinicalTrial.gov website under the identification number NCT01675999. This study received a grant from the French National Cancer Institute (INCA) in 2010. The study complies with the Declaration of Helsinki rules and the principles of the Good Clinical Practices guidelines. Informed consent will be obtained from each patient in a written form prior to randomisation. Patient safety and all potential threats to the patients will be monitored every 6 months by an independent data safety monitoring board (DSMB) and, additionally, at the discretion of the DSMB or Promoter. The DSMB also will evaluate the primary endpoint data. Qualified personnel at the sponsor site will also meet every three months to review safety data, including adverse events and serious adverse events. Any information deemed to potentially affect the safety of the trial will be brought to the attention of the DSMB. An Independent committee (IDMC) including at least 1 clinicien, 1 methodologist and pharmacologist will be created before trial initiation. This IDMC should statute on efficacy and safety at the interim analysis regarding decision criteria of Simon design, post operative morbidity.

## Discussion

There are several arguments in favor of performing a study to assess the impact on survival of perioperative chemotherapy in the treatment of stage II/III CC. Three prerequisites are however essential. First, systemic chemotherapy must be effective and able to induce a measurable and reproducible response of the primary tumor. Second, current preoperative imaging with abdominopelvic CT scan must allow for an accurate assessment of tumor stage to justify antineoplastic treatment with a limited number of false-positives patients in order not to over-treat patients that do not require such treatments. Finally, the neoadjuvant chemotherapy must be well tolerated and not increase the risk of pre- and post-operative complications.

### Tumor Histopathological response to chemotherapy

The histopathological changes induced by neoadjuvant chemotherapy and/or radiotherapy have been studied in several digestive cancers, such as cancer of the esophagus, stomach, rectum and liver metastases of colorectal origin [19, 23–27]. In these cancers, histopathological changes included increased fibrosis, the appearance of acellular necrosis, inflammatory infiltration, the appearance of colloid pools and the reduction or disappearance of tumor cells. However, no grading system for histopathological response has been validated to date [22, 27, 28]. In CC, the "local" effect of systemic chemotherapy on the primary tumor has long been suspected based on indirect arguments from patients receiving chemotherapy for metastatic disease without resection of the primary tumor, such as resolution of clinical symptoms and radiological changes. In a pilot study [15], we characterized the histopathological changes and assessed the response of primary colon tumors among patients with metastatic CC treated with chemotherapy first and subsequently resected. The grading system for the histopathological tumor regression was based on that of Mandard and collaborators [22], derived from the study of tumor response after chemoradiation in squamous cell cancer of the esophagus, then adapted by Rubbia-Brandt and colleagues [27] for the regression of liver metastases of colorectal origin. In our study, when compared with a control group, neoadjuvant chemotherapy was associated with extensive fibrosis and marked lymphoplasmacytic infiltration in nearly half the cases, and changes in the mucosa overlying the tumor (normal colonic mucosa or inflammatory ulceration) in 75 % of cases. Using the Tumor Regression Grade (TRG), nearly 70 % of CCs treated with neoadjuvant chemotherapy showed signs of major histological response characterized by fibrosis outgrowing residual cancer cells. The histological response in the primary tumor was correlated to that observed in liver metastases for 9 patients operated on for both colon and liver metastases after chemotherapy [15].

### Selection of patients with locally advanced CC by CT scan

Hundt and colleagues reported on the sensitivity of helical CT in imaging colorectal cancer in 37 patients [29]. Arterial and portal phases were examined. The overall sensitivity for detecting cancer was 97 % in both phases, and 86 % in the portal phase alone. The sensitivity for the detection of lymph node involvement when correlated with pathological data was 67.6 %, with 23 node positive patients detected out of a total of 34. The positive predictive value of CT (1 lymph node greater than 1 cm, or more than 3 lymph nodes less than 1 cm in diameter with a maximum enhancement greater than 100 HU) was 95.4 %. The overall accuracy of CT compared with pathologic stage was 81 %. Of the 18 T1 or T2 tumors, only two were incorrectly classified as T3 based on the scans. For the 19 T3 or T4 tumors, only two were incorrectly classified as T2.

More recently, Burton and colleagues sought to investigate if the CT scan could elucidate poor prognostic factors for CC in order to select a subset of patients eligible for neoadjuvant treatment [30]. Three patients were imaged by a single shot spiral CT with a 10 mm collimation and a pitch of 1. The portal phase was scanned, after the upper gastrointestinal tract was opacified with water. Returning to the criteria set forth in the literature to distinguish T0-T2 from T3-T4 tumors, the authors proposed that CT could highlight poor prognostic factors (extension to adjacent organs, extension greater than 5 mm in to the peritoneal fat, lymph node involvement) that correlated with pathological data. Of 14 tumors determined to be T3 on pathology, 10 (71.4 %) were correctly classified as T3 by CT, one was incorrectly upstaged to T4 by CT (7.1 %), and 3 were incorrectly down-staged by CT (21 %). For the 12 pathologically determined T1 and T2 tumors, between 3 and 5 tumors were classified as T3 by CT. Finally, of the seven T4 tumors, between one and two tumors were classified as either T0 or T2 based on CT. Regarding lymph node status, the presence of 1 to 3 positive nodes greater than 1 cm, or the grouping of more than 3 positive nodes less than 3 mm in short axis, or the grouping of more than 3 positive nodes more than 1 cm in short axis (N2) provided an accuracy of 64 % for lymph node staging. These results were comparable to those previously reported in other studies [31]. According to radiologists, there was an over-staging in 24 to 30 % of cases. By exploiting the radiological data of this study, Smith and colleagues [32] compared groups of patients with and without poor prognostic factors by CT scan. Disease-free survival at 3 years was 71 % in the group with a good prognosis CT versus 43 % in the poor prognosis CT group (p <0.0066). These results were similar to those based on pathologic staging, with 75 % versus 43 % survival at 3 years. The authors concluded that CT staging could differentiate between patients with good and poor colorectal cancer prognosis. These data were recently confirmed in a series of 94 patients recruited from 18 centers [14].

### The choice of neoadjuvant chemotherapy regimens

FOLFOX-4 is the standard of care treatment given postoperatively for 6 months in patients with stage III CC [4]. In advanced disease, the effectiveness of FOLFOX-4 has been demonstrated in terms of response rate, progression-free survival and overall survival, with good patient tolerability [33–38]. In advanced disease with resectable liver metastases, the efficacy of perioperative chemotherapy with FOLFOX-4 (6 cycles before and 6 cycles after surgery) was demonstrated in a prospective, randomized trial [18]. In this study of 364 patients with 1 to 4 resectable liver metastases, a 9.2 % benefit in progression-free survival at 3 years was observed in the treatment arm (33.2 % [25.3-41.2] vs. 42.4 % [34.0-50.5], HR 0.73 [0.55-0.97], p = 0.025) at the cost of increased postoperative morbidity (25 % vs. 15.9 %, p = 0.04) and more biliary complications. In this study, 37 of 171 patients (39 %) treated with neoadjuvant FOLFOX-4 had a partial or complete radiographic response by RECIST criteria.

The combination of FOLFOX-4 and Cetuximab has also been demonstrated to be effective with regard to response rate (57 to 81 %) and progression-free survival with good tolerance, in patients with non-mutated *KRAS* (wild-type) metastatic colorectal cancer [39–46]. Two Phase III trials comparing FOLFOX-4 and FOLFOX-4 plus Cetuximab after resection of stage III CC have demonstrated no benefit when adding cetuximab. In the US NCCTG NO147 trial, the three-year disease-free survival for mFOLFOX6 alone was 74.6 % vs. 71.5 % with the addition of cetuximab (HR, 1.21; 95 % CI, 0.98-1.49; P = .08) in 1863 patients with wild-type KRAS. In the European PETACC8 trial including 2564 patients, the 3-year disease-free survival in the 1602 KRAS WT patients was 78 % in the FOLFOX arm and 75 % in the cetuximab arm (p = 0.652). However though both trials are negatives, and didn’t show any benefit of adding cetuximab to FOLFOX in term of DFS for resected stage III CC, no detrimental effect of the FOLFOX + cetuximab combination therapy was observed even in patients with KRAS mutated tumors. The use of this combination therapy in the neoadjuvant setting, in RAS WT patients, remains to our opinion an attractive option for two reasons: the high reported response rates observed with this schedule in metastatic patients (57 to 81 %) with a manageable toxicity profile and the significant increase in disease-free survival when adding cetuximab in the subgroup of patients operated from a locally advanced (pT4N2) tumor reported in the PETACC8 trial.

The aim of our phase II ECKINOXE study is to evaluate the efficacy (response rate) and tolerability of two regimens of neoadjuvant chemotherapy among patients with locally advanced CC. The control arm will consist of patients treated with FOLFOX-4 post-operatively. This study will allow us to investigate the feasibility and effectiveness of this original neo-adjuvant strategy, and to select the best regimen to improve long-term disease-free and overall survivals. A similar study testing another anti-EGFR monoclonal antibody (Panitumumab) is currently ongoing in the United Kingdom (FOxTROT trial). The pilot stage of this randomised controlled trial on the first 150 included has been recently published. Nearly 90 % (85 of 95) of patients in this trial completed preoperative chemotherapy with grade 3–4 gastrointestinal toxicity in 7 %. All 99 tumours in the preoperative group were resected, with no significant differences in postoperative morbidity between the preoperative and control groups. T3 or more advanced tumours was pathologically confirmed in 98 % of patients undergoing immediate surgery and 91 % of patients following preoperative chemotherapy (p = 0 · 10). Preoperative therapy resulted in significant downstaging in terms of TNM stage compared with the postoperative group (p = 0 · 04), including two pathological complete responses.
